# Molecular Dynamics Simulations: Advances and Applications

**DOI:** 10.3390/molecules27072105

**Published:** 2022-03-24

**Authors:** Hugo A. L. Filipe, Luís M. S. Loura

**Affiliations:** 1 Coimbra Chemistry Center, Institute of Molecular Sciences (CQC-IMS), University of Coimbra, 3004-535 Coimbra, Portugal; 2 CPIRN-IPG, Center of Potential and Innovation of Natural Resources, Polytechnic Institute of Guarda, 6300-559 Guarda, Portugal; 3 CNC, Center for Neuroscience and Cell Biology, 3004-504 Coimbra, Portugal; 4 Faculty of Pharmacy, University of Coimbra, 3000-548 Coimbra, Portugal

Molecular dynamics (MD) simulations have led to great advances in many scientific disciplines, such as chemical physics, materials science, and biophysics. This computational methodology has demonstrated high relevance in the detailed characterization of biomolecular systems, including complementarity with experimental data, experimental design optimization, and prediction of relevant properties for chemical systems that are expensive or difficult to handle experimentally. Among many applications, it has been employed to characterize disease development processes and has been used in the initial stages of drug design and development. The application of MD simulations to the characterization of biomolecular systems is very broad, encompassing the characterization of membrane structure and organization, membrane permeability, lipid–protein interactions, lipid–drug interactions, protein–ligand interactions, and protein structure and dynamics. The articles published in this Special Issue illustrate the versatility of MD simulations in this context.

In this Special Issue, Cardoso et al. analyzed the inhibition of tyrosinase activity using kojic acid (KA) derivatives designed from aromatic aldehydes and malononitrile [1]. Tyrosinases belong to the functional copper-containing proteins family and the dysregulation of their activity is involved in skin cancer initiation. MD simulations revealed that the derivatives formed promising complexes with tyrosinase, suggesting that these derivatives could be potent competitive inhibitors of the natural substrates. Hernández-Ochoa et al. tested 55 compounds to gain insights into their possible use as new inhibitory drugs of *Helicobacter pylori* glucose-6-phosphate dehydrogenase (HpG6PD) activity [2]. *H. pylori* is a pathogen that can remain in the stomach of an infected person for their entire life, potentially leading to the development of severe gastric diseases such as gastric cancer. The in silico study of the chemical compounds discovered possible interactions with the HpG6PD enzyme, finding compounds that can be internalized at the NADP+ catalytic binding site with the probability to exert a competitive inhibitory effect on NADP+ and a non-competitive or uncompetitive effect on HpG6PD. The tested compounds inhibiting HpG6PD represent promising novel drug candidates against *H. pylori*. Al-Thiabat et al. addressed drugs targeting folate receptor alpha (FRα), a biological marker in cancer drug delivery [3]. Folic acid (FA) was conjugated to beta-cyclodextrin (βCD) and subjected to in silico analysis by molecular docking and MD simulations to investigate the affinity and stability of the conjugated system compared to unconjugated and apo systems (ligand-free). Analyses using MD simulations with root-mean-square deviation (RMSD), root-mean-square fluctuation (RMSF), and radius of gyration (Rg) demonstrated that FA and FA–βCDs created more dynamically stable systems with FRα than the apo-FRα system. Protein residues that might have a direct role in increasing the stability of holo systems were identified. Lui et al. used MD simulations to explore the hydration dynamics of staphylococcal nuclease (SNase) at different temperatures and mutation levels [4]. The dynamics of protein–water fluctuations are of biological significance, and it was described how the hydrogen bonding relaxation governed local protein fluctuations. The structural and dynamical properties of protein–water at the molecular level are described as fundamental to the physiological and functional mechanisms of SNase. Yao et al. used MD simulations to study mitochondrial ADP/ATP carrier (AAC) transport mechanisms of ATP export and ADP import [5]. In this study, all-atom MD simulations were employed on a variety of mutants and the CATR-AAC complex, revealing a detailed description of this transport mechanism. The results provide new insights into the highly conserved yet variable m-gate network in the big mitochondrial carrier family.

Addressing the topic of MD simulations of lipid membrane systems, Magalhães et al. described the interactions of rhodamine dyes, widely used as fluorescent tags in cell imaging and as P-glycoprotein model substrates [6]. The detailed understanding of the interaction between different rhodamine species and biomembranes was obtained, combining atomistic MD simulations and fluorescence spectroscopy. Subtle distinctions in the interaction with POPC membranes were found among different ionization forms of the probes. Membrane-spanning free-energy profiles were compared with lipid/water partition coefficients. This work provided detailed insights into the similarities and differences in the behavior of bilayer-inserted Rhodamines 123 and B, which are related to the structure of the probes. The much higher affinity to the membranes of the latter increases the local concentration and explains its higher apparent affinity for P-glycoprotein reconstituted in model membranes.

Finally, on the topic of force-field comparison and development, Patmanidis et al. used MD simulations in conjunction with metadynamics to calculate the free energy of dimerization of small aromatic rings, and compared three models from popular online servers for atomistic force fields, namely G54a7, CHARMM36 and OPLS [7]. Dimerization free energies are fundamental quantities that describe the strength of interactions of different molecules. It was shown that regardless of the force field, the profiles for the dimerization free energy of these compounds are very similar. Larger challenges appear for studying larger molecules, resulting in force-field-dependent preferred stacking modes, where significant care needs to be taken.
